# A *HGF* Mutation in the Familial Case of Primary Lymphedema: A Report

**DOI:** 10.3390/ijms25105464

**Published:** 2024-05-17

**Authors:** Galina Koksharova, Natalia Kokh, Maria Gridina, Rustam Khapaev, Vadim Nimaev, Veniamin Fishman

**Affiliations:** 1 Institute of Cytology and Genetics, Lavrentyev Prospekt, 10, 630090 Novosibirsk, Russia; g.koksharova@g.nsu.ru (G.K.);; 2 Department of Natural Sciences, Novosibirsk State University, 2 Pirogov Street, 630090 Novosibirsk, Russia; 3 Institute of Chemical Biology and Fundamental Medicine, 630090 Novosibirsk, Russia; 4 Research Institute of Clinical and Experimental Lymphology, Branch of the Institute of Cytology and Genetics, Timakova Street, 2, 630060 Novosibirsk, Russia

**Keywords:** primary lymphedema, Meige disease, whole-exome sequencing, HGF

## Abstract

Lymphedema is a disorder that leads to excessive swelling due to lymphatic insufficiency, resulting in the accumulation of protein-rich interstitial fluid. Primary lymphedema predominantly impacts the lower extremities and is frequently linked to hereditary factors. This condition is known to be associated with variants in several genes, such as *FOXC2*, *FLT4*, and *SOX18*. However, many cases remain unexplained, suggesting undiscovered gene associations. This study describes a novel mutation in the hepatocyte growth factor (*HGF*) gene, a previously hypothesized candidate for lymphedema pathogenesis. This mutation was identified in affected members of a multigenerational family presenting with primary leg lymphedema, consistent with an autosomal dominant inheritance pattern.

## 1. Introduction

Lymphedema is a type of edema (or swelling) that is caused by lymphatic dysfunction. It is categorized into two types: primary lymphedema, which is thought to be a hereditary condition, and secondary lymphedema, which arises from injury or obstruction of lymphatic nodes or, more rarely, lymphatic collectors.

Primary lymphedema can be an indicator of various complex hereditary syndromes, such as Noonan syndrome (associated with the *PTPN11* gene) [1], lymphedema–distichiasis syndrome (*FOXC2*) [2], and hypotrichosis–lymphedema–telangiectasia syndrome (*SOX18*) [3]. Another common lymphedema condition is Milroy disease (*FLT4*) [4], characterized as congenital onset lymphedema, mostly affecting the legs [5]. However, not all lymphedema disorders have a clear genetic link. One such case is Meige disease, a form of late-onset primary lymphedema typically presenting with isolated lesions in the lower limbs. While mutations in the *GJC2* gene have been observed in some familial cases of Meige disease [5], a significant proportion of cases do not have a known gene association, highlighting the need for further genetic research in the area.

Generally, it is estimated that identifiable genetic variants are present in only about 30% of primary lymphedema cases [6]. Because of this, there is an ongoing search for novel gene associations. Among the potential candidates, mutations in the *HGF* (hepatocyte growth factor) gene are particularly noteworthy.

*HGF* encodes a protein that binds to the hepatocyte growth factor receptor, playing a role in regulating cell growth. Previous research, including in vitro studies and in vivo experiments on mice, has demonstrated that HGF protein binding promotes lymphangiogenesis, a critical process in the development of the lymphatic system [7]. Another study showed that *HGF* expression via plasmid transfer ameliorates secondary lymphedema in the rat tail injury model [8].

*HGF* is also linked to other genes integral to lymphedema’s molecular–genetic mechanisms, for example, *VEGFR-2*, which is known for activating angiogenesis [9]. Additionally, genes such as *KDR*, *BMPR2*, *SERPINE1*, *IL10*, and *CCL2*, which have been identified as possible candidates for lymphedema through the Associative Network Discovery System, are associated with *HGF* [10].

Various mutations in the *HGF* gene have been documented in lymphedema patients (all reported variants are summarized in Table 1). One study [11] reported four cases, with three involving primary lymphedema patients and one with lymphedema and lymphangiectasia. Another study [12] described four additional cases in patients presenting with congenital or late-onset lymphedema in sporadic and familial cases. However, these studies provided limited information on the clinical manifestations of the disease. Later research [13] reported 12 variants in 11 patients who presented with primary lymphedema, mostly of the lower extremities, with a variable age of onset and no additional dysmorphic features. The *HGF* mutations identified were predominantly loss-of-function or missense variants affecting the HGF SP domain, crucial for receptor interaction [14]. Interestingly, it was noticed [13] that loss-of-function mutations tended to result in a more severe phenotype. However, none of these reports provide sufficient details to unambiguously confirm the causative role of *HGF* mutations in lymphedema development. Nevertheless, the growing number of cases has led some clinics to include *HGF* mutation screening in their diagnostic protocols [15].

To contribute to this area of study, in this report, we describe a novel loss-of-function *HGF* mutation identified in a multigenerational family with primary lymphedema.

## 2. Materials and Methods

### 2.1. Patients

**Figure 1:**
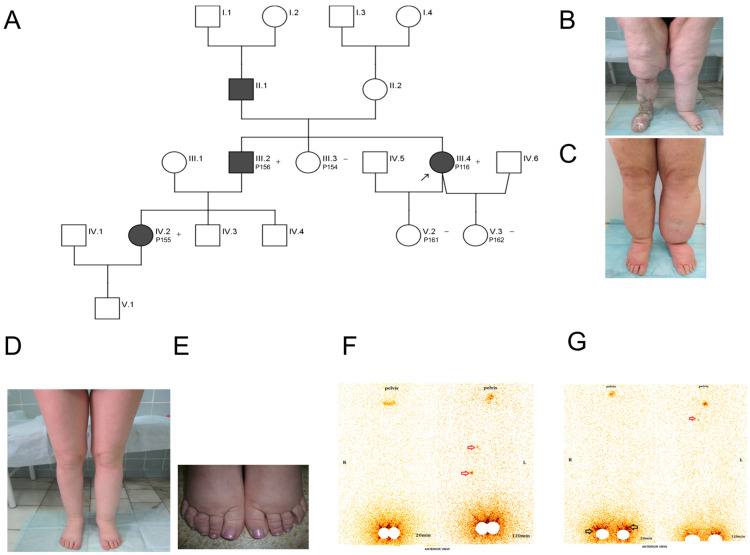
Family with primary lymphedema. (**A**) A pedigree displaying the familial relationships among patients. An arrow points at patient P116, for whom NGS analysis was performed. P154, P155, P156, P161, and P162 were tested for the presence of HGF:p.(Arg533Ter), which was found in P116. The presence of the variant in a heterozygous state is denoted by “+”, while its absence is indicated by “−”. (**B**) A photograph of patient P116 illustrating her condition of primary bilateral leg lymphedema (taken after previous resection surgery in the shin and foot). (**C**) Phenotype of P116’s brother, P156, who exhibited asymmetrical edema. (**D**,**E**) Phenotype of P116’s niece, P155, presenting with asymmetrical oedema and papillomatosis of the fingers. (**F**) Lymphoscintigram of P155. No lymphatic channels or inguinal lymph nodes were visualized on both sides. The local delay of the radiotracer resulting from recruitment of the deep lymphatics due to insufficiency of the superficial lymphatics is shown with red arrows. (**G**) Lymphoscintigram of P156. No lymphatic channels were visualized on both sides. The absence of uptake in the ilioinguinal nodes on the left side is visible. Bilateral dermal backflow in the lower limbs in the region of the ankles is shown with black arrows. The low-intensity visualization of the inguinal lymph node on the right side is shown with a red arrow.

A family with a history of primary lymphedema was consulted at the clinic of the Research Institute of Clinical and Experimental Lymphology—Branch Institute of Cytology and Genetics SB RAS Novosibirsk, Russia (Figure 1A; affected members are shown in black).

### 2.2. Whole-Exome Sequencing

Genetic testing was performed on germline DNA extracted from blood. NGS data for patient P116 was obtained using the exome sequencing protocol with the Roche HyperExome panel.

For our analysis, we primarily searched for variants in the following list of genes associated with lymphedema and lymphatic disease [16]:

*ACVRL1*, *AKT1*, *ARAF*, *ARHGAP31*, *BRAF*, *CBL*, *CCBE1*, *CCM2*, *CELSR1*, *CTNNB1*, *DCHS1*, *ELMO2*, *ENG*, *EPHB4*, *FAT4*, *FGFR1*, *FLT4*, *FOXC2*, *GATA2*, *GDF2*, *GJC2*, *GLMN*, *GNA11*, *GNA14*, *GNAQ*, *HGF*, *HRAS*, *IDH1*, *IDH2*, *KIF11*, *KRAS*, *KRIT1*, *MAP2K1*, *MAP2K2*, *MAP3K1*, *MAP3K3*, *MAPK1*, *MAPK14*, *MAPK3*, *MET*, *MTOR*, *NRAS*, *PDCD10*, *PDGFRB*, *PIEZO1*, *PIK3CA*, *PTEN*, *PTPN11*, *PTPN14*, *RAF1*, *RASA1*, *RIT1*, *SHOC2*, *SMAD4*, *SOS1*, *SOX18*, *STAMBP*, *TEK*, *TP53*, and *VEGFC*.

### 2.3. Segregation Analysis

For the segregation analysis of the variant in the *HGF* gene identified through NGS, a specific strategy was employed. Primers were selected to amplify the 591 bp segment of the *HGF* gene, which included the variant. Then, a restriction analysis was performed using the FaeI enzyme.

The variant of interest introduces a recognition site for the FaeI enzyme, resulting in distinguishable digestion products with lengths of 341 and 250 bp. In contrast, the reference allele does not have a FaeI site and thus presents as a single band of 591 bp after digestion.

## 3. Results and Discussion

### 3.1. Clinical Manifestations of the Familial Lymphedema Case

We performed a genetic analysis of the primary lymphedema patient, P116.

P116 is a 75-year-old woman presenting with primary bilateral leg lymphedema, classified as Stage III according to the International Society of Lymphology staging system (Figure 1B). She has been affected since the age of 9, when she first received the diagnosis of lymphedema.

Her medical history includes multiple interventions and comorbidities. At the age of 17, the patient underwent the Charles procedure on the right tibia and foot and later the excision of papillomas on the right foot at the age of 49. Additionally, she has been diagnosed with left-sided gonarthrosis, morbid obesity (with a body mass undex of 41.6 kg/m^2^), hypertension, left ventricular hypertrophy, dyslipidemia, ischemic heart disease, subclinical hypothyroidism, nephroangiosclerosis, and stage 2 chronic kidney disease (as indicated by a Chronic Kidney Disease Epidemiology Collaboration score of 56 and a Cockcroft–Gault calculation of 67 mL/min/1.73 m^2^). She also has a history of chronic calculous cholecystitis, which is currently in remission.

P116 has a family history of lymphedema with multiple affected relatives (see Figure 1A for a pedigree).

As evident from Figure 1A, the pattern of inheritance in the family is autosomal dominant. Common clinical features of all affected patients in the family include recurrent erysipelas of the lower extremities and limited swelling of the legs. This swelling is typically confined to the area below the knee, including the feet, and can be asymmetrical in nature. None of the affected individuals have distichiasis, hair, skin, or any additional vascular abnormalities.

Affected relatives that we have more data on include P156 and P155, a brother and a niece of P116. They developed lymphedema of both lower limbs at the ages of 12 and 17, respectively. However, their clinical manifestations vary. P155 exhibits less pronounced symptoms compared to P116 and P156 (see Figure 1B–E). Her condition has remained relatively stable without the need for complex decongestive therapy (CDT), relying instead on compression hosiery and weight management. This stability might be attributed to better medical care and early intervention. Still, recent lymphoscintigraphy of the legs performed on P155 showed severe lymphatic drainage dysfunction of the lower limbs, despite the fact that she underwent microsurgical interventions involving anastomosis between transected inguinal lymph nodes and a large vein [17]. A similar lymphatic drainage impairment is observed in her father, P156, as evident from the lymphoscintigram of his lower limbs (Figure 1F,G).

### 3.2. Identification of the HGF Variant in the Familial Lymphedema Case

For the genetic analysis of this family, we sequenced the exome of patient P116. We specifically looked for variation in the genes associated with lymphedema and lymphatic disease, including the most well-researched ones like *FOXC2*, *SOX18*, and *FLT4*.

We identified a heterozygous mutation, HGF:p.(Arg533Ter). This mutation is a single nucleotide substitution (chr7:81707309G>A, hg19) that introduces a premature STOP codon, causing a truncation of the protein. It is located in the SP domain of the HGF protein, which is the domain responsible for binding to the HGF receptor [18]. This is consistent with previous reports that mostly describe mutations perturbing HGF domains important for interaction with the receptor [11,12,13]. Still, by ACMG/AMP guidelines, the variant is not considered pathogenic. Franklin [19] gives it an automatic rating of VUS (leaning LP).

After identifying HGF:p.(Arg533Ter) in patient P116 through NGS data, we performed a segregation analysis on the patient’s family members in an attempt to confirm the variant’s significance. We observed that the variant cosegregated with the disease (Figure 1A and Scheme S1), which further supports the connection between HGF:p.(Arg533Ter) and lymphedema.

Interestingly, other studies also show a connection between *HGF* and erysipelas, which is a recurrent feature in the affected individuals in this family. It was demonstrated [20] that patients carrying the *SOD2* C2734T mutation, which is associated with an increased susceptibility to erysipelas, had lower serum HGF levels compared to those with the wild-type *SOD2* gene. This may hint at the link between *HGF* and erysipelas, but a lot more data is needed to support that link.

In conclusion, the association between the *HGF* gene and primary lymphedema is gaining more support with emerging case studies like the one we are reporting. This particular case is notable for several reasons. It is the first one describing the HGF:p.(Arg533Ter) mutation and also the first one to include a large multigenerational family with the *HGF* mutation, as opposed to the cases reported previously [11,13], where only first-degree relatives of the patients were tested. Another important aspect of this case study is the comprehensive description of the clinical manifestations of lymphedema in the affected individuals. Providing such detailed clinical profiles is crucial for enhancing our understanding of the variable presentations of lymphedema and its impact on patients.

Still, it is important to note that there is no definitive proof for the causative role of *HGF* variants (and HGF:p.(Arg533Ter) specifically) in primary lymphedema, as no functional studies have been conducted. The accumulating cases, however, do suggest a possible connection, highlighting the need for further research. Future studies, especially those involving functional analyses and larger patient cohorts, will be critical in determining the role of *HGF* in primary lymphedema and potentially guiding targeted therapeutic approaches.

## Figures and Tables

**Table 1:** A list of *HGF* variants reported in previous studies, complete with an associated condition and family segregation data.

*HGF* Mutation	Associated Condition	Segregation in the Family	Reported by
p.(Cys288*)	primary lymphedema	no data	Finegold et al. [11]
p.(Arg630*)	lymphedema and lymphangiectasia	no data	
p.(Val631Met)	primary lymphedema	found in at least one affected first-degree relative of the proband	
p.(Leu634Phe)	primary lymphedema	found in at least one affected first-degree relative of the proband	
p.(Arg178*)	primary lymphedema	no data	Michelini et al. [12]
p.(Arg424Cys)	primary lymphedema	no data	
p.(Trp451Arg)	primary lymphedema	no data	
p.(Lys491Asnfs*6)	primary lymphedema	no data	
p.Cys96*	primary lymphedema	no data	Mengeot [13]
p.Met385Asnfs*	primary lymphedema	no data	
p.Arg502*	primary lymphedema	found in one non-affected first-degree relative of the proband	
p.Gly557*	primary lymphedema	no data	
p.Gly618Alafs*	primary lymphedema	no data	
c.1444+1G>A	primary lymphedema	found in one affected first-degree relative of the proband	
p.Pro187Thr	primary lymphedema	no data	
p.Val509Ile	primary lymphedema	not found in one non-affected first-degree relative of the proband	
p.Thr605Arg	primary lymphedema	found in one non-affected first-degree relative of the proband	
p.Asn624Lys	primary lymphedema	located in trans; non-affected parents carry different variants	
p.Gly627Asp			
p.Lys649Asn	primary lymphedema	found in one non-affected first-degree relative of the proband	

## Data Availability

Raw human sequencing data are not available due to the personal data sharing policy. Other data analyzed in this study are available from the corresponding author on reasonable request.

## Supplementary Materials

The supporting information can be downloaded at: https://www.mdpi.com/article/10.3390/ijms25105464/s1.

## References

[1] Tartaglia M., Mehler E.L., Goldberg R., Zampino G., Brunner H.G., Kremer H., van der Burgt I., Crosby A.H., Ion A., Jeffery S. (2001). Mutations in PTPN11, Encoding the Protein Tyrosine Phosphatase SHP-2, Cause Noonan Syndrome. Nat. Genet..

[2] Fang J., Dagenais S.L., Erickson R.P., Arlt M.F., Glynn M.W., Gorski J.L., Seaver L.H., Glover T.W. (2000). Mutations in FOXC2 (MFH-1), a Forkhead Family Transcription Factor, Are Responsible for the Hereditary Lymphedema-Distichiasis Syndrome. Am. J. Hum. Genet..

[3] Irrthum A., Devriendt K., Chitayat D., Matthijs G., Glade C., Steijlen P.M., Fryns J.-P., Steensel M.A.M.V., Vikkula M. (2003). Mutations in the Transcription Factor Gene SOX18 Underlie Recessive and Dominant Forms of Hypotrichosis-Lymphedema-Telangiectasia. Am. J. Hum. Genet..

[4] Irrthum A., Karkkainen M.J., Devriendt K., Alitalo K., Vikkula M. (2000). Congenital Hereditary Lymphedema Caused by a Mutation That Inactivates VEGFR3 Tyrosine Kinase. Am. J. Hum. Genet..

[5] Gordon K., Varney R., Keeley V., Riches K., Jeffery S., Van Zanten M., Mortimer P., Ostergaard P., Mansour S. (2020). Update and audit of the St George’s classification algorithm of primary lymphatic anomalies: A clinical and molecular approach to diagnosis. J. Med. Genet..

[6] Sung C., Wang S., Hsu J., Yu R., Wong A.K. Current Understanding of Pathological Mechanisms of Lymphedema|Advances in Wound Care. https://www.liebertpub.com/doi/abs/10.1089/wound.2021.0041.

[7] Kajiya K., Hirakawa S., Ma B., Drinnenberg I., Detmar M. (2005). Hepatocyte Growth Factor Promotes Lymphatic Vessel Formation and Function. EMBO J..

[8] Saito Y., Nakagami H., Morishita R., Takami Y., Kikuchi Y., Hayashi H., Nishikawa T., Tamai K., Azuma N., Sasajima T. (2006). Transfection of Human Hepatocyte Growth Factor Gene Ameliorates Secondary Lymphedema via Promotion of Lymphangiogenesis. Circulation.

[9] Sulpice E., Ding S., Muscatelli-Groux B., Bergé M., Han Z.C., Plouet J., Tobelem G., Merkulova-Rainon T. Cross-Talk between the VEGF-A and HGF Signalling Pathways in Endothelial Cells—Sulpice—2009—Biology of the Cell—Wiley Online Library. https://onlinelibrary.wiley.com/doi/full/10.1042/BC20080221.

[10] Saik O.V., Nimaev V.V., Usmonov D.B., Demenkov P.S., Ivanisenko T.V., Lavrik I.N., Ivanisenko V.A. Prioritization of Genes Involved in Endothelial Cell Apoptosis by Their Implication in Lymphedema Using an Analysis of Associative Gene Networks with ANDSystem|BMC Medical Genomics. https://link.springer.com/article/10.1186/s12920-019-0492-9.

[11] Finegold D.N., Schacht V., Kimak M.A., Lawrence E.C., Foeldi E., Karlsson J.M., Baty C.J., Ferrell R.E. HGF and MET Mutations in Primary and Secondary Lymphedema|Lymphatic Research and Biology. https://www.liebertpub.com/doi/abs/10.1089/lrb.2008.1524.

[12] Michelini S., Vettori A., Maltese P.E., Cardone M., Bruson A., Fiorentino A., Cappellino F., Sainato V., Guerri G., Marceddu G. (2016). Genetic screening in a large cohort of italian patients affected by primary lymphedema using a next generation sequencing (NGS) approach. Lymphology.

[13] Mengeot L., Revencu N., Vikkula M. Primary Lymphedema and HGF Mutation: Analysis of the Phenotype in 11 Patients with Primary Lymphedema Associated with Mutation in HGF Gene & Review of Literature|Mémoire UCL. https://dial.uclouvain.be/memoire/ucl/en/object/thesis%3A13430.

[14] Holmes O., Pillozzi S., Deakin J.A., Carafoli F., Kemp L., Butler P.J.G., Lyon M., Gherardi E. (2007). Insights into the Structure/Function of Hepatocyte Growth Factor/Scatter Factor from Studies with Individual Domains. J. Mol. Biol..

[15] Gordon K., Mortimer P.S., van Zanten M., Jeffery S., Ostergaard P., Mansour S. The St George’s Classification Algorithm of Primary Lymphatic Anomalies|Lymphatic Research and Biology. https://www.liebertpub.com/doi/10.1089/lrb.2020.0104?url_ver=Z39.88-2003&rfr_id=ori%3Arid%3Acrossref.org&rfr_dat=cr_pub++0pubmed.

[16] Seo S.H., Lee S., Park J.K.-H., Yang E.J., Kim B., Lee J.-S., Kim M.J., Park S.S., Seong M.-W., Nam S.-Y. (2022). Clinical Staging and Genetic Profiling of Korean Patients with Primary Lymphedema Using Targeted Gene Sequencing. Sci. Rep..

[17] Pak C.S., Suh H.P., Kwon J.G., Cho M.-J., Hong J.P. (2021). Lymph Node to Vein Anastomosis (LNVA) for Lower Extremity Lymphedema. J. Plast. Reconstr. Aesthet. Surg..

[18] Tahira Y., Sakai K., Sato H., Imamura R., Matsumoto K. (2021). Dimer Interface in Natural Variant NK1 Is Dispensable for HGF-Dependent Met Receptor Activation. Int. J. Mol. Sci..

[19] Genoox F. (2021). The Future of Genomic Medicine. https://franklin.genoox.com/clinical-db/home.

[20] Emene C.C., Kravchenko I.E., Aibatova G.I., Albert A.R. Analysis of Serum Cytokines and Single-Nucleotide Polymorphisms of SOD1, SOD2, and CAT in Erysipelas Patients. https://www.hindawi.com/journals/jir/2017/2157247/.

