# Supplementary material for: A HGF Mutation in the Familial Case of Primary Lymphedema: A Report
Source: Int J Mol Sci. 2024 May 17;25(10):5464. doi: 10.3390/ijms25105464 (PMC11122351; doi:10.3390/ijms25105464)
Supplement: Supplementary file 1 [file ijms-25-05464-s001.zip › ijms-2980372-supplementary.pdf]

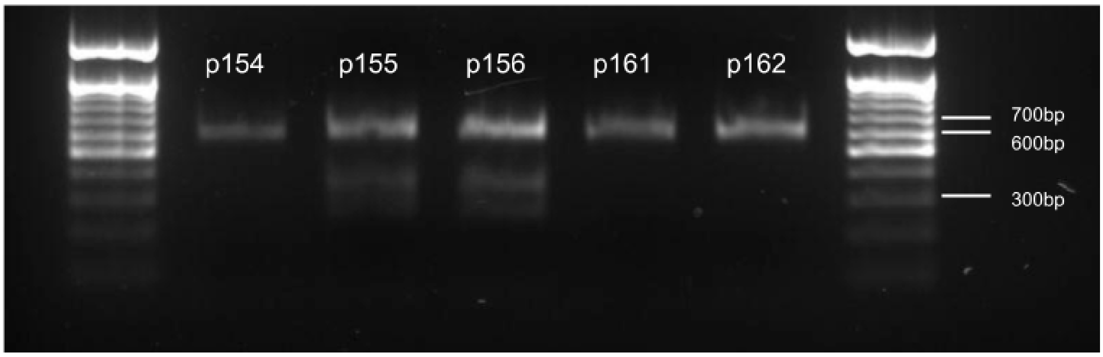

Scheme S1. Results of the segregation analysis of HGF:p.(Arg533Ter) in the relatives of P116. Restriction analysis was performed on the 591 bp sequence of HGF gene containing the variant, amplified using PCR. These PCR products were digested with FaeI restriction enzyme: if the variant of interest was present, a FaeI recognition site would be generated in the sequence resulting in digestion products with lengths 341 bp and 250 bp. Meanwhile, the reference allele does not have FaeI site and thus presents as a single band of 591 bp after digestion.
